# Guanidinium l-glutamate

**DOI:** 10.1107/S1600536810036354

**Published:** 2010-09-30

**Authors:** Bing Peng, Qingrong Peng, Wenfeng Zhou, Zhiqiang Zhou

**Affiliations:** a Department of Applied Chemistry, China Agricultural University, Yuanmingyuan, West Road 2, Haidian District, Beijing 100194, People’s Republic of China

## Abstract

In the title compound, CH_6_N_3_^+^·C_5_H_8_NO_4_^−^, there are two independent cations and two independent anions in the asymmetric unit. In the crystal structure, cations and anions are linked by inter­molecular N—H⋯O hydrogen bonds into a three-dimensional network.

## Related literature

For an early report of salts formed from amino acids and guanidines, see: Armstrong (1956 ▶).
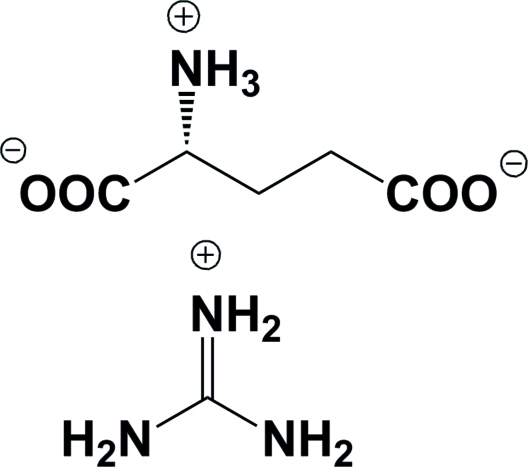


## Experimental

### 

#### Crystal data

CH_6_N_3_^+^·C_5_H_8_NO_4_^−^
*M* *_r_* = 206.21
Monoclinic, 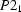

*a* = 8.7793 (7) Å
*b* = 10.8729 (10) Å
*c* = 10.0801 (9) Å
β = 104.552 (1)°
*V* = 931.34 (14) Å^3^
*Z* = 4
Mo *K*α radiation
μ = 0.12 mm^−1^
*T* = 150 K
0.42 × 0.26 × 0.20 mm

#### Data collection

Bruker SMART APEX diffractometer
Absorption correction: multi-scan (*SADABS*; Sheldrick, 1996 ▶) *T*_min_ = 0.950, *T*_max_ = 0.976
5501 measured reflections
2220 independent reflections
2087 reflections with *I* > 2σ(*I*)
*R*_int_ = 0.021

#### Refinement

*R*[*F* ^2^ > 2σ(*F* ^2^)] = 0.031
*wR*(*F* ^2^) = 0.081
*S* = 1.06
2220 reflections
255 parameters
1 restraint
H-atom parameters constrained
Δρ_max_ = 0.30 e Å^−3^
Δρ_min_ = −0.23 e Å^−3^

### 

Data collection: *SMART* (Bruker, 1997 ▶); cell refinement: *SAINT* (Bruker, 1997 ▶); data reduction: *SAINT*; program(s) used to solve structure: *SHELXTL* (Sheldrick, 2008 ▶); program(s) used to refine structure: *SHELXTL*; molecular graphics: *PLATON* (Spek, 2009 ▶); software used to prepare material for publication: *SHELXTL*.

## Supplementary Material

Crystal structure: contains datablocks I, global. DOI: 10.1107/S1600536810036354/lh5125sup1.cif

Structure factors: contains datablocks I. DOI: 10.1107/S1600536810036354/lh5125Isup2.hkl

Additional supplementary materials:  crystallographic information; 3D view; checkCIF report

## Figures and Tables

**Table 1:** Hydrogen-bond geometry (Å, °)

*D*—H⋯*A*	*D*—H	H⋯*A*	*D*⋯*A*	*D*—H⋯*A*
N1—H1*A*⋯O8	0.91	1.89	2.795 (2)	179
N1—H1*B*⋯O4^i^	0.91	1.84	2.738 (2)	170
N1—H1*C*⋯O2^i^	0.91	2.13	3.017 (2)	165
N2—H2*A*⋯O2^ii^	0.91	2.09	2.998 (2)	173
N2—H2*B*⋯O7^iii^	0.91	2.16	2.740 (2)	120
N2—H2*C*⋯O5^iii^	0.91	1.92	2.817 (3)	170
N3—H3*A*⋯O2^i^	0.88	2.08	2.900 (3)	154
N3—H3*B*⋯O3	0.88	2.08	2.841 (3)	145
N4—H4*A*⋯O3^iv^	0.88	1.95	2.826 (2)	173
N4—H4*B*⋯O1^i^	0.88	2.22	3.095 (2)	170
N5—H5*A*⋯O4^iv^	0.88	1.96	2.831 (2)	172
N5—H5*B*⋯O6	0.88	2.35	3.092 (3)	142
N6—H6*A*⋯O6	0.88	2.04	2.897 (2)	165
N6—H6*B*⋯O8^v^	0.88	1.97	2.824 (2)	164
N7—H7*A*⋯O5	0.88	2.00	2.851 (2)	163
N7—H7*B*⋯O8^vi^	0.88	2.02	2.775 (3)	143
N8—H8*A*⋯O7^v^	0.88	2.08	2.954 (3)	170
N8—H8*B*⋯O1^vi^	0.88	2.23	2.953 (3)	140

Symmetry codes: (i) 
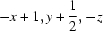
; (ii) 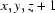; (iii) 
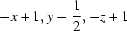
; (iv) 
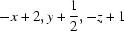
; (v) 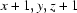; (vi) 
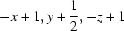
.

**Fig. 1.**
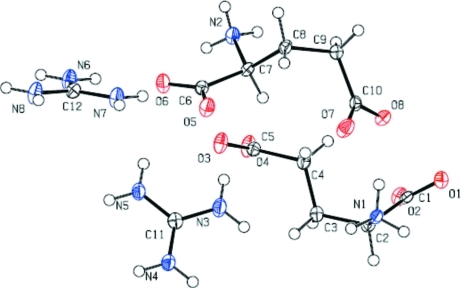
The asymmetric unit of the title compound with displacement ellipsoids drawn at the 30% probability level.

**Fig. 2.**
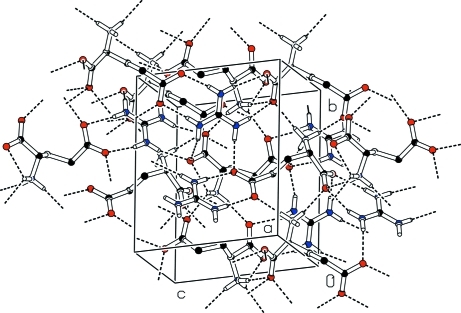
Part of the crystal structure of the title compound with hydrogen bonds shown as dashed lines.

## supplementary crystallographic information

### Comment

To better understand the formation of complex salts between a guanidine compounds and amino acids we carried out the crystal structure determination of the title compound. The asymmetric unit of the title compound is shown in Fig. 1. There are two independent cations and two indpendent anions in the asymmetric unit. In the crystal structure, cations and anions are linked by intramolecular N—H···O hydrogen bonds into a three-dimensional network (see Fig. 2).

### Experimental

L-Glutamic acid (1.47 g.) and guanidine carbonate (0.90 g) were suspended in 10 ml of water. When the evolution of CO_2_ had ceased the solution was diluted with 20 ml of acetone, and evaporated to a clear syrup. The syrup was dissolved in 30 ml of absolute methanol to yield a clear solution, and was allowed to stand overnight at room temperature. This solution was then placed in a fume hood for another day, whereupon the crystals of the title compound were collected and dried.

### Refinement

In the absence of significant anomalous dispersion effects Friedel pairs were merged. The absolute configuation is known from the starting material. H atoms were placed in calculated positions (C—H = 0.99 or 1.00 Å, N—H = 0.88 or 0.91 Å) and were refined as riding, with *U*_iso_(H) = 1.2*U*_eq_(C,*N*) or 1.5_eq_(N) for –NH_3_ groups.

### Crystal data

**Table:**

CH_6_N_3_^+^·C_5_H_8_NO_4_^−^	*F*(000) = 440
*M**_r_* = 206.21	*D*_x_ = 1.471 Mg m^−^^3^
Monoclinic, *P*2_1_	Mo *K*α radiation, λ = 0.71073 Å
Hall symbol: P 2yb	Cell parameters from 2748 reflections
*a* = 8.7793 (7) Å	θ = 2.4–27.5°
*b* = 10.8729 (10) Å	µ = 0.12 mm^−^^1^
*c* = 10.0801 (9) Å	*T* = 150 K
β = 104.552 (1)°	Prism, colourless
*V* = 931.34 (14) Å^3^	0.42 × 0.26 × 0.20 mm
*Z* = 4	

### Data collection

**Table:**

Bruker SMART APEX diffractometer	2220 independent reflections
Radiation source: fine-focus sealed tube	2087 reflections with *I* > 2σ(*I*)
graphite	*R*_int_ = 0.021
φ and ω scans	θ_max_ = 27.5°, θ_min_ = 2.4°
Absorption correction: multi-scan (*SADABS*; Sheldrick, 1996)	*h* = −11→10
*T*_min_ = 0.950, *T*_max_ = 0.976	*k* = −14→9
5501 measured reflections	*l* = −8→13

### Refinement

**Table:**

Refinement on *F*^2^	Primary atom site location: structure-invariant direct methods
Least-squares matrix: full	Secondary atom site location: difference Fourier map
*R*[*F*^2^ > 2σ(*F*^2^)] = 0.031	Hydrogen site location: inferred from neighbouring sites
*wR*(*F*^2^) = 0.081	H-atom parameters constrained
*S* = 1.06	*w* = 1/[σ^2^(*F*_o_^2^) + (0.044*P*)^2^ + 0.2149*P*] where *P* = (*F*_o_^2^ + 2*F*_c_^2^)/3
2220 reflections	(Δ/σ)_max_ < 0.001
255 parameters	Δρ_max_ = 0.30 e Å^−^^3^
1 restraint	Δρ_min_ = −0.22 e Å^−^^3^

### Special details

**Table:**

Geometry. All e.s.d.'s (except the e.s.d. in the dihedral angle between two l.s. planes) are estimated using the full covariance matrix. The cell e.s.d.'s are taken into account individually in the estimation of e.s.d.'s in distances, angles and torsion angles; correlations between e.s.d.'s in cell parameters are only used when they are defined by crystal symmetry. An approximate (isotropic) treatment of cell e.s.d.'s is used for estimating e.s.d.'s involving l.s. planes.
Refinement. Refinement of *F*^2^ against ALL reflections. The weighted *R*-factor *wR* and goodness of fit *S* are based on *F*^2^, conventional *R*-factors *R* are based on *F*, with *F* set to zero for negative *F*^2^. The threshold expression of *F*^2^ > σ(*F*^2^) is used only for calculating *R*-factors(gt) *etc*. and is not relevant to the choice of reflections for refinement. *R*-factors based on *F*^2^ are statistically about twice as large as those based on *F*, and *R*- factors based on ALL data will be even larger.

### Fractional atomic coordinates and isotropic or equivalent isotropic displacement parameters (Å^2^)

**Table:**

	*x*	*y*	*z*	*U*_iso_*/*U*_eq_	
C1	0.3633 (2)	0.4498 (2)	−0.1328 (2)	0.0157 (4)	
C2	0.4808 (2)	0.5516 (2)	−0.06507 (19)	0.0152 (4)	
H2	0.5234	0.5892	−0.1389	0.018*	
C3	0.6207 (2)	0.5030 (2)	0.0442 (2)	0.0175 (4)	
H3C	0.6924	0.5723	0.0803	0.021*	
H3D	0.6793	0.4434	0.0016	0.021*	
C4	0.5727 (2)	0.4405 (2)	0.1632 (2)	0.0185 (4)	
H4C	0.4937	0.3761	0.1264	0.022*	
H4D	0.5227	0.5020	0.2111	0.022*	
C5	0.7121 (2)	0.3823 (2)	0.2661 (2)	0.0161 (4)	
C6	0.6284 (3)	0.5883 (2)	0.6253 (2)	0.0170 (4)	
C7	0.5001 (2)	0.5088 (2)	0.5315 (2)	0.0142 (4)	
H7	0.5153	0.5159	0.4368	0.017*	
C8	0.3304 (2)	0.5466 (2)	0.52340 (19)	0.0164 (4)	
H8C	0.3259	0.6367	0.5357	0.020*	
H8D	0.2954	0.5067	0.5990	0.020*	
C9	0.2181 (2)	0.5106 (2)	0.3863 (2)	0.0171 (4)	
H9A	0.2379	0.4240	0.3655	0.020*	
H9B	0.1083	0.5165	0.3946	0.020*	
C10	0.2368 (2)	0.5920 (2)	0.2679 (2)	0.0156 (4)	
C11	0.9131 (2)	0.7443 (2)	0.4492 (2)	0.0176 (4)	
C12	0.9193 (3)	0.7368 (2)	0.9641 (2)	0.0179 (4)	
N1	0.3944 (2)	0.65067 (18)	−0.01100 (17)	0.0155 (4)	
H1A	0.3351	0.6164	0.0415	0.023*	
H1B	0.3307	0.6918	−0.0823	0.023*	
H1C	0.4648	0.7040	0.0407	0.023*	
N2	0.5301 (2)	0.37747 (18)	0.57395 (19)	0.0183 (4)	
H2A	0.4960	0.3633	0.6507	0.027*	
H2B	0.6351	0.3617	0.5918	0.027*	
H2C	0.4774	0.3275	0.5052	0.027*	
N3	0.7857 (2)	0.6972 (2)	0.3634 (2)	0.0240 (4)	
H3A	0.7411	0.7361	0.2870	0.029*	
H3B	0.7461	0.6272	0.3832	0.029*	
N4	0.9733 (2)	0.84922 (19)	0.42024 (19)	0.0204 (4)	
H4A	1.0578	0.8797	0.4770	0.024*	
H4B	0.9288	0.8887	0.3441	0.024*	
N5	0.9797 (2)	0.68433 (19)	0.5639 (2)	0.0215 (4)	
H5A	1.0643	0.7145	0.6209	0.026*	
H5B	0.9394	0.6144	0.5829	0.026*	
N6	0.9603 (2)	0.62410 (19)	0.9389 (2)	0.0242 (4)	
H6A	0.9036	0.5832	0.8682	0.029*	
H6B	1.0442	0.5899	0.9926	0.029*	
N7	0.7924 (2)	0.78798 (19)	0.88260 (19)	0.0222 (4)	
H7A	0.7361	0.7467	0.8121	0.027*	
H7B	0.7648	0.8631	0.8991	0.027*	
N8	1.0012 (2)	0.8000 (2)	1.0716 (2)	0.0248 (5)	
H8A	1.0844	0.7668	1.1274	0.030*	
H8B	0.9721	0.8751	1.0868	0.030*	
O1	0.22034 (18)	0.47085 (14)	−0.15334 (15)	0.0197 (3)	
O2	0.42560 (19)	0.35290 (15)	−0.16612 (15)	0.0206 (3)	
O3	0.76473 (19)	0.43703 (16)	0.37806 (16)	0.0221 (4)	
O4	0.76678 (19)	0.28334 (16)	0.23297 (16)	0.0236 (4)	
O5	0.6030 (2)	0.70163 (15)	0.62702 (17)	0.0234 (4)	
O6	0.75073 (18)	0.53379 (16)	0.68837 (16)	0.0225 (4)	
O7	0.2657 (2)	0.70271 (16)	0.28796 (17)	0.0277 (4)	
O8	0.21550 (17)	0.54149 (15)	0.15054 (14)	0.0184 (3)	

### Atomic displacement parameters (Å^2^)

**Table:**

	*U*^11^	*U*^22^	*U*^33^	*U*^12^	*U*^13^	*U*^23^
C1	0.0218 (10)	0.0148 (10)	0.0098 (9)	−0.0013 (8)	0.0027 (8)	0.0026 (8)
C2	0.0177 (9)	0.0146 (10)	0.0126 (8)	−0.0017 (8)	0.0025 (7)	−0.0002 (8)
C3	0.0153 (9)	0.0193 (10)	0.0165 (10)	0.0003 (9)	0.0013 (8)	−0.0003 (8)
C4	0.0159 (10)	0.0222 (12)	0.0161 (10)	0.0029 (9)	0.0017 (8)	−0.0005 (9)
C5	0.0153 (9)	0.0163 (10)	0.0158 (10)	0.0001 (8)	0.0024 (8)	0.0010 (8)
C6	0.0180 (10)	0.0187 (11)	0.0135 (9)	−0.0030 (9)	0.0024 (8)	0.0002 (8)
C7	0.0177 (9)	0.0134 (10)	0.0110 (9)	0.0014 (8)	0.0023 (7)	0.0009 (8)
C8	0.0167 (9)	0.0203 (10)	0.0115 (9)	0.0013 (8)	0.0024 (7)	0.0006 (8)
C9	0.0166 (9)	0.0184 (10)	0.0151 (9)	−0.0024 (8)	0.0021 (7)	0.0000 (8)
C10	0.0126 (9)	0.0167 (10)	0.0155 (10)	0.0014 (8)	−0.0005 (7)	0.0014 (8)
C11	0.0169 (10)	0.0180 (11)	0.0182 (10)	0.0029 (8)	0.0048 (8)	−0.0029 (8)
C12	0.0186 (10)	0.0176 (11)	0.0169 (10)	0.0009 (8)	0.0035 (8)	0.0015 (8)
N1	0.0186 (8)	0.0142 (8)	0.0122 (8)	−0.0007 (7)	0.0008 (6)	0.0010 (7)
N2	0.0202 (9)	0.0136 (9)	0.0184 (9)	0.0008 (7)	−0.0003 (7)	−0.0010 (7)
N3	0.0246 (10)	0.0203 (10)	0.0214 (9)	−0.0026 (8)	−0.0052 (8)	0.0023 (8)
N4	0.0205 (9)	0.0211 (10)	0.0172 (9)	−0.0025 (8)	0.0001 (7)	0.0001 (8)
N5	0.0189 (9)	0.0211 (10)	0.0206 (9)	−0.0030 (8)	−0.0024 (7)	0.0020 (8)
N6	0.0237 (10)	0.0209 (11)	0.0233 (10)	0.0065 (8)	−0.0028 (8)	−0.0031 (8)
N7	0.0240 (10)	0.0171 (9)	0.0209 (9)	0.0043 (8)	−0.0032 (8)	−0.0035 (8)
N8	0.0284 (10)	0.0203 (11)	0.0195 (9)	0.0056 (8)	−0.0057 (8)	−0.0024 (8)
O1	0.0183 (7)	0.0212 (9)	0.0175 (7)	−0.0006 (6)	0.0010 (6)	−0.0013 (6)
O2	0.0253 (8)	0.0183 (8)	0.0167 (8)	0.0026 (7)	0.0024 (6)	−0.0035 (6)
O3	0.0235 (8)	0.0218 (9)	0.0171 (8)	0.0053 (7)	−0.0020 (6)	−0.0046 (7)
O4	0.0253 (8)	0.0208 (8)	0.0199 (8)	0.0068 (7)	−0.0032 (6)	−0.0048 (7)
O5	0.0280 (9)	0.0137 (8)	0.0232 (8)	−0.0024 (7)	−0.0033 (7)	0.0003 (6)
O6	0.0191 (8)	0.0201 (9)	0.0234 (8)	0.0000 (7)	−0.0035 (6)	−0.0009 (7)
O7	0.0405 (10)	0.0178 (8)	0.0195 (8)	−0.0069 (8)	−0.0025 (7)	0.0026 (7)
O8	0.0220 (7)	0.0185 (8)	0.0143 (7)	0.0015 (6)	0.0039 (6)	0.0016 (6)

### Geometric parameters (Å, °)

**Table:**

C1—O1	1.241 (3)	C10—O7	1.237 (3)
C1—O2	1.271 (3)	C10—O8	1.275 (3)
C1—C2	1.549 (3)	C11—N4	1.321 (3)
C2—N1	1.497 (3)	C11—N5	1.328 (3)
C2—C3	1.524 (3)	C11—N3	1.332 (3)
C2—H2	1.0000	C12—N6	1.319 (3)
C3—C4	1.527 (3)	C12—N7	1.329 (3)
C3—H3C	0.9900	C12—N8	1.331 (3)
C3—H3D	0.9900	N1—H1A	0.9100
C4—C5	1.529 (3)	N1—H1B	0.9100
C4—H4C	0.9900	N1—H1C	0.9100
C4—H4D	0.9900	N2—H2A	0.9100
C5—O4	1.257 (3)	N2—H2B	0.9100
C5—O3	1.257 (3)	N2—H2C	0.9100
C6—O6	1.251 (3)	N3—H3A	0.8800
C6—O5	1.253 (3)	N3—H3B	0.8800
C6—C7	1.541 (3)	N4—H4A	0.8800
C7—N2	1.495 (3)	N4—H4B	0.8800
C7—C8	1.528 (3)	N5—H5A	0.8800
C7—H7	1.0000	N5—H5B	0.8800
C8—C9	1.533 (3)	N6—H6A	0.8800
C8—H8C	0.9900	N6—H6B	0.8800
C8—H8D	0.9900	N7—H7A	0.8800
C9—C10	1.527 (3)	N7—H7B	0.8800
C9—H9A	0.9900	N8—H8A	0.8800
C9—H9B	0.9900	N8—H8B	0.8800
			
O1—C1—O2	126.4 (2)	C10—C9—H9B	109.1
O1—C1—C2	118.4 (2)	C8—C9—H9B	109.1
O2—C1—C2	115.21 (18)	H9A—C9—H9B	107.8
N1—C2—C3	112.11 (16)	O7—C10—O8	123.2 (2)
N1—C2—C1	109.42 (16)	O7—C10—C9	119.6 (2)
C3—C2—C1	113.44 (18)	O8—C10—C9	117.14 (19)
N1—C2—H2	107.2	N4—C11—N5	120.2 (2)
C3—C2—H2	107.2	N4—C11—N3	120.4 (2)
C1—C2—H2	107.2	N5—C11—N3	119.4 (2)
C2—C3—C4	112.99 (17)	N6—C12—N7	119.7 (2)
C2—C3—H3C	109.0	N6—C12—N8	121.3 (2)
C4—C3—H3C	109.0	N7—C12—N8	119.0 (2)
C2—C3—H3D	109.0	C2—N1—H1A	109.5
C4—C3—H3D	109.0	C2—N1—H1B	109.5
H3C—C3—H3D	107.8	H1A—N1—H1B	109.5
C3—C4—C5	112.67 (17)	C2—N1—H1C	109.5
C3—C4—H4C	109.1	H1A—N1—H1C	109.5
C5—C4—H4C	109.1	H1B—N1—H1C	109.5
C3—C4—H4D	109.1	C7—N2—H2A	109.5
C5—C4—H4D	109.1	C7—N2—H2B	109.5
H4C—C4—H4D	107.8	H2A—N2—H2B	109.5
O4—C5—O3	124.40 (19)	C7—N2—H2C	109.5
O4—C5—C4	117.95 (18)	H2A—N2—H2C	109.5
O3—C5—C4	117.6 (2)	H2B—N2—H2C	109.5
O6—C6—O5	126.2 (2)	C11—N3—H3A	120.0
O6—C6—C7	116.6 (2)	C11—N3—H3B	120.0
O5—C6—C7	117.06 (19)	H3A—N3—H3B	120.0
N2—C7—C8	111.79 (18)	C11—N4—H4A	120.0
N2—C7—C6	108.12 (16)	C11—N4—H4B	120.0
C8—C7—C6	115.78 (18)	H4A—N4—H4B	120.0
N2—C7—H7	106.9	C11—N5—H5A	120.0
C8—C7—H7	106.9	C11—N5—H5B	120.0
C6—C7—H7	106.9	H5A—N5—H5B	120.0
C7—C8—C9	112.17 (17)	C12—N6—H6A	120.0
C7—C8—H8C	109.2	C12—N6—H6B	120.0
C9—C8—H8C	109.2	H6A—N6—H6B	120.0
C7—C8—H8D	109.2	C12—N7—H7A	120.0
C9—C8—H8D	109.2	C12—N7—H7B	120.0
H8C—C8—H8D	107.9	H7A—N7—H7B	120.0
C10—C9—C8	112.69 (17)	C12—N8—H8A	120.0
C10—C9—H9A	109.1	C12—N8—H8B	120.0
C8—C9—H9A	109.1	H8A—N8—H8B	120.0
			
O1—C1—C2—N1	11.3 (3)	O6—C6—C7—N2	18.9 (3)
O2—C1—C2—N1	−170.89 (17)	O5—C6—C7—N2	−163.9 (2)
O1—C1—C2—C3	137.29 (19)	O6—C6—C7—C8	145.18 (19)
O2—C1—C2—C3	−44.9 (2)	O5—C6—C7—C8	−37.6 (3)
N1—C2—C3—C4	63.7 (2)	N2—C7—C8—C9	−83.4 (2)
C1—C2—C3—C4	−60.8 (2)	C6—C7—C8—C9	152.21 (18)
C2—C3—C4—C5	174.99 (19)	C7—C8—C9—C10	−73.5 (2)
C3—C4—C5—O4	−75.2 (3)	C8—C9—C10—O7	−36.4 (3)
C3—C4—C5—O3	104.4 (2)	C8—C9—C10—O8	146.12 (19)

### Hydrogen-bond geometry (Å, °)

**Table:**

*D*—H···*A*	*D*—H	H···*A*	*D*···*A*	*D*—H···*A*
N1—H1A···O8	0.91	1.89	2.795 (2)	179
N1—H1B···O4^i^	0.91	1.84	2.738 (2)	170
N1—H1C···O2^i^	0.91	2.13	3.017 (2)	165
N2—H2A···O2^ii^	0.91	2.09	2.998 (2)	173
N2—H2B···O7^iii^	0.91	2.16	2.740 (2)	120
N2—H2C···O5^iii^	0.91	1.92	2.817 (3)	170
N3—H3A···O2^i^	0.88	2.08	2.900 (3)	154
N3—H3B···O3	0.88	2.08	2.841 (3)	145
N4—H4A···O3^iv^	0.88	1.95	2.826 (2)	173
N4—H4B···O1^i^	0.88	2.22	3.095 (2)	170
N5—H5A···O4^iv^	0.88	1.96	2.831 (2)	172
N5—H5B···O6	0.88	2.35	3.092 (3)	142
N6—H6A···O6	0.88	2.04	2.897 (2)	165
N6—H6B···O8^v^	0.88	1.97	2.824 (2)	164
N7—H7A···O5	0.88	2.00	2.851 (2)	163
N7—H7B···O8^vi^	0.88	2.02	2.775 (3)	143
N8—H8A···O7^v^	0.88	2.08	2.954 (3)	170
N8—H8B···O1^vi^	0.88	2.23	2.953 (3)	140

Symmetry codes: (i) −*x*+1, *y*+1/2, −*z*; (ii) *x*, *y*, *z*+1; (iii) −*x*+1, *y*−1/2, −*z*+1; (iv) −*x*+2, *y*+1/2, −*z*+1; (v) *x*+1, *y*, *z*+1; (vi) −*x*+1, *y*+1/2, −*z*+1.
